# Multilayered Insights into Poorly Differentiated, *BRAFV600E*-Positive, Thyroid Carcinoma in a Rapidly Developing Goiter with Retrosternal Extension: From En “Y” Cervicotomy to SPECT/CT-Positive Lung Metastases

**DOI:** 10.3390/diagnostics15162049

**Published:** 2025-08-15

**Authors:** Oana-Claudia Sima, Anca-Pati Cucu, Dana Terzea, Claudiu Nistor, Florina Vasilescu, Lucian-George Eftimie, Mihai-Lucian Ciobica, Mihai Costachescu, Mara Carsote

**Affiliations:** 1 Department of Clinical Endocrinology V, “C.I. Parhon” National Institute of Endocrinology, 011863 Bucharest, Romania; oana-claudia.sima@drd.umfcd.ro (O.-C.S.); carsote_m@hotmail.com (M.C.); 2 PhD Doctoral School, “Carol Davila” University of Medicine and Pharmacy, 010825 Bucharest, Romania; anca-pati.cucu@drd.umfcd.ro; 3 Thoracic Surgery Department, “Dr. Carol Davila” Central Emergency University Military Hospital, 010825 Bucharest, Romania; mihai.costachescu@drd.umfcd.ro; 4 Department of Pathology, “C.I. Parhon” National Institute of Endocrinology, 011863 Bucharest, Romania; danaterzea@gmail.com; 5 Department 4—Cardio-Thoracic Pathology, Thoracic Surgery II Discipline, “Carol Davila” University of Medicine and Pharmacy, 0505474 Bucharest, Romania; 6 Department of Pathology, “Dr. Carol Davila” Central Emergency University Military Hospital, 010825 Bucharest, Romania; florinapath@yahoo.com (F.V.); lucian.eftimie@unefs.ro (L.-G.E.); 7 Department of Internal Medicine and Gastroenterology, “Carol Davila” University of Medicine and Pharmacy, 020021 Bucharest, Romania; 8 Department of Internal Medicine I and Rheumatology, “Dr. Carol Davila” Central Military University Emergency Hospital, 010825 Bucharest, Romania; 9 Department of Radiology and Medical Imaging, “Dr. Carol Davila” Central Emergency University Military Hospital, 010825 Bucharest, Romania; 10 Department of Endocrinology, “Carol Davila” University of Medicine and Pharmacy, 020021 Bucharest, Romania

**Keywords:** thyroid malignancy, SPECT/CT, scintigraphy, neck ultrasound, contrast-enhanced computed tomography, thyroidectomy, thyroid malignancy, thoracic surgery, TSH, thyroglobulin, iodine, gene, goiter, RAS, P53

## Abstract

Poorly differentiated thyroid malignancy, a rare histological type of aggressive thyroid malignancy with associated difficulties and gaps in its histological and molecular characterization, might lead to challenging clinical presentations that require a prompt multimodal approach. This case study involved a 56-year-old, non-smoking male with a rapidly developing goiter (within 2–3 months) in association with mild, non-specific neck compressive symptoms. His medical history was irrelevant. A voluminous goiter with substernal and posterior extension up to the vertebral bodies was detected using an ultrasound and computed tomography (CT) scan and required emergency thyroidectomy. He had normal thyroid function, as well as negative thyroid autoimmunity and serum calcitonin. The surgery was successful upon “Y” incision, which was used to give better access to the retrosternal component in order to avoid a sternotomy. Post-operatively, the subject developed hypoparathyroidism-related hypocalcemia and showed a very high serum thyroglobulin level (>550 ng/mL). The pathological report confirmed poorly differentiated, multifocal thyroid carcinoma (with an insular, solid, and trabecular pattern) against a background of papillary carcinoma (pT3b, pN0, and pM1; L1; V2; Pn0; R1; and stage IVB). The subject received 200 mCi of radioiodine therapy for 6 weeks following the thoracic surgery. Whole-body scintigraphy was performed before radioiodine therapy and showed increased radiotracer uptake at the thyroid remnants and pre-tracheal levels. Additionally, single-photon emission computed tomography combined with CT (SPECT/CT) was performed, and confirmed the areas of intense uptake, in addition to a moderate uptake in the right and left pulmonary parenchyma, suggesting lung metastasis. To conclude, an overall low level of statistical evidence exists regarding poorly differentiated malignancy in substernal goiters, and the data also remains scarce regarding the impact of genetic and molecular configurations, such as the *BRAF*-positive profile, in this specific instance. Furthermore, multimodal management includes additional diagnosis methods such as SPECT/CT, while long-term multilayered therapy includes tyrosine kinase inhibitors if the outcome shows an iodine-resistant profile with a poor prognosis. Awareness remains a key factor in cases of a poorly differentiated carcinoma presenting as a rapidly growing goiter with substernal extension in an apparently healthy adult. A surgical approach, while varying with the surgeon’s skills, represents a mandatory step to ensure a better prognosis. In addition to a meticulous histological characterization, genetic/molecular features provide valuable information regarding the outcome and can further help with the decision to use new anti-cancer drugs if tumor response upon radioiodine therapy is no longer achieved; such a development is expected in this disease stage in association with a *BRAF*-positive configuration.

**Figure 1:**
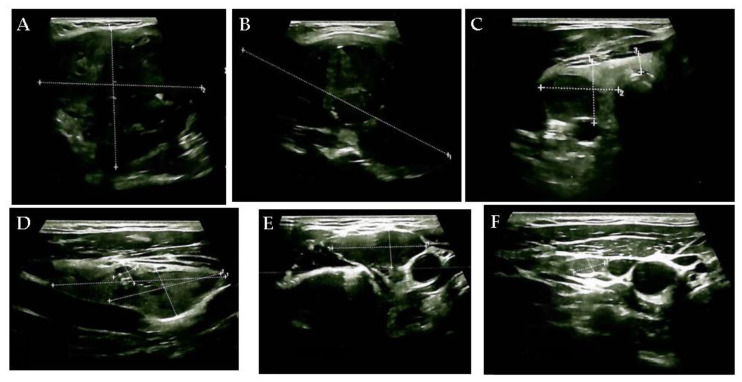
This case study involved a 56-year-old, non-smoking male with a rapidly developing goiter (within 2–3 months) without neck compressive symptoms (the neck mass was detected by self-palpation). His medical history was irrelevant; he was not diagnosed with any endocrine condition, and he was not taking any chronic medication. The patient was living in a non-endemic (non-iodine-deficient) geographic area. A normal biochemical and hormonal baseline panel, including thyroid function and negative thyroid autoimmunity and calcitonin (Table S1), was associated with anterior neck ultrasound features revealing an enlarged, multinodular goiter: the right thyroid lobe (of 2.1 by 2.5 by 5.5 cm) and the left lobe (of 5.75 by 6 by 10 cm) had a hypoechoic, inhomogeneous and multinodular pattern. The left lobe was displaced by a large hypoechoic, inhomogeneous nodular conglomerate of 6 by 8 by 10 cm ((**A**). transverse plane) with micro-calcifications and macro-calcifications ((**B**). longitudinal plane), causing tracheal deviation. The right lobe also presented a hypoechoic nodule of 3.7 by 1.6 by 1.9 cm, with increased vascularity and necrosis ((**C**). transverse plane) and another hypoechoic nodule of 0.75 by 0.7 by 0.7 cm ((**D**). longitudinal plane). The upper left latero-cervical region showed a hypoechoic, inhomogeneous, highly vascularized lymph node enlargement of 3 by 1.4 cm ((**E**). transversal plane), and multiple lymph nodes, sized between 0.3 and 1.8 cm. Five other lymph nodes with a hypoechoic aspect, the largest being 0.9 by 0.5 cm in the middle third part ((**F**) the transverse plane), were found in the right latero-cervical area.

**Figure 2:**
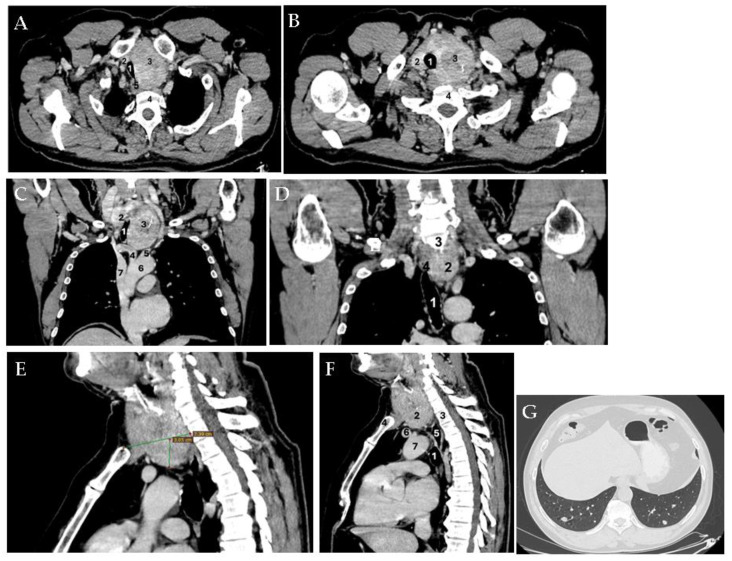
Since there was no hyperthyroidism, intravenous contrast-enhanced computed tomography (CT) was performed, which confirmed the voluminous goiter with posterior extension towards the vertebral bodies. ((**A**). Axial plane: 1—compressed trachea; 2—right thyroid lobe; 3—left thyroid lobe; 4—cervical spine; 5—deviated esophagus. (**B**). Axial plane: 1—deviated trachea; 2—right thyroid lobe; 3—left thyroid lobe; 4—cervical spine). The left internal jugular vein and the left carotid artery were displaced to the left (without a cleavage plane from these vascular structures). ((**C**). Coronal plane: 1—compressed trachea; 2—right thyroid lobe; 3—left thyroid lobe; 4—brachiocephalic trunk; 5—left common carotid artery; 6—aortic arch; 7—superior cave vein. (**D**). Coronal plane: 1—deviated trachea; 2—thyroid mass; 3—C7 cervical vertebra; 4—deviated esophagus). The thyroid mass caudally extended on the left paratracheal side, reaching the upper mediastinum in contact with the trachea, deviating it to the right and narrowing it to a diameter of 1.53 by 2.66 cm. It also caused the left lateral deviation of the mediastinal vessels. ((**E**). Sagittal plane: The space occupying the mass in the left thyroid lobe and isthmus with a 3.05 cm extension (vertical line) from the superior thoracic aperture (horizontal line) into the mediastinum is shown. (**F**). Sagittal plane: 1—trachea; 2—left thyroid lobe; 3—T2 thoracic vertebra corresponding to the lower pole of the tumor; 4—manubrium; 5—esophagus; 6—left venous brachiocephalic trunk; 7—aortic arch). The goiter had various diameters at different sections; for instance, at the level of the cricoid cartilage, diameters were 4.84 by 5.14 cm, respectively. At the clavicular level, diameters were 7 by 7.26 cm. The isthmus protruded at the level of the jugular notch (of 3 by 4.4 cm), which is situated para-tracheal on the left and posterior to the manubrium of 4.22 by 4.82 cm (the longitudinal extension reached up to 10 cm cranio-caudally). This large substernal goiter was iodophilic and showed a heterogeneous structure both on non-contrast and contrast-enhanced CT phases, while also revealing micro-calcifications and macro-calcifications. Moreover, the patient presented multiple lung micro-nodules, which were disseminated bilaterally. ((**G**). Lung window; axial plane). The abdominal and cerebral CT scans were normal.

**Figure 3:**
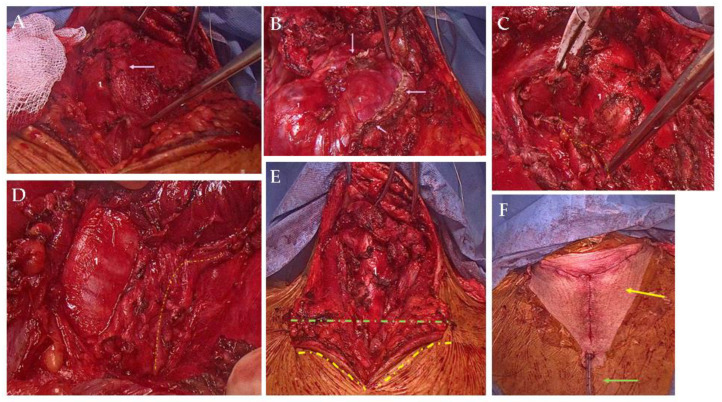
Considering the tracheal deviation of the large goiter with retrosternal presentation, an emergency thyroidectomy was performed in the Thoracic Surgery Department. A “Y” cervicotomy was preferred in order to provide optimum access for a radical resection. The cervical component of the “Y” incision was deepened beyond the platysma, where the dissection was performed at this level in the superior, inferior, and lateral directions. Any bleeding was controlled with LigaSure®. Crossing anterior jugular veins were sealed and divided with LigaSure®. The deep cervical fascia was divided longitudinally, and the strap muscles were lifted off the right and left thyroid lobes. The left thyroid lobe was enlarged with subsequent right tracheal shifting and compression; the left lower pole descended retrosternally. (Intraoperative findings: (**A**). 1—enlarged thyroid lobe underneath the strap muscle layers, with pink arrow-growth invasion of the left strap muscles. (**B**). 1—enlarged thyroid lobe, with pink arrow at the margins of resected strap muscles). The right middle thyroid vein was identified by the gentle retraction of the thyroid towards the midline. The vessels were sealed and divided with LigaSure®. The superior thyroid vascular bundle was dissected, sealed, and divided with care so as not to injure the external branch of the superior laryngeal nerve and the recurrent laryngeal nerve, followed by the inferior vascular bundle, which was then dissected, sealed, and divided. Both the right upper and right lower parathyroid glands were visualized and preserved. The left middle vascular bundle was identified, sealed, and divided. The left thyroid lobe was enlarged and descended retrosternally. The upper left vascular bundle was dissected, and both the external branch of the superior laryngeal nerve and the recurrent laryngeal nerve were found and preserved. The upper left pole thyroid vascular bundle was then sealed and divided; next, the inferior pole was approached. Since the CT imaging showed no vascular bundle for the left lobe originating from the mediastinal vessels, cervical delivery of the left lobe was possible. ((**C**,**D**): 1—trachea; yellow dotted line—right recurrent laryngeal nerve running parallel to the trachea at different operating times). Hemostasis was achieved using LigaSure®. A cervical drain tube was placed in the left neck thyroid bed. The strap muscle and the platysma were approximated with 3-0 Vicryl® sutures. The skin was re-approximated using subcuticular stitches with 4-0 Vicryl® and Steri-Strip® bandages. The patient tolerated the procedure well and was extubated in the operating room and taken to the post-operative unit. [(**E**). 1—trachea: green-dotted line—Kocher incision; yellow dotted line—the lateral margins of the vertical supplementary incision. (**F**). Yellow arrow—neck “Y” incision (en “Y” cervicotomy); green arrow—neck drain tube].

**Figure 4:**
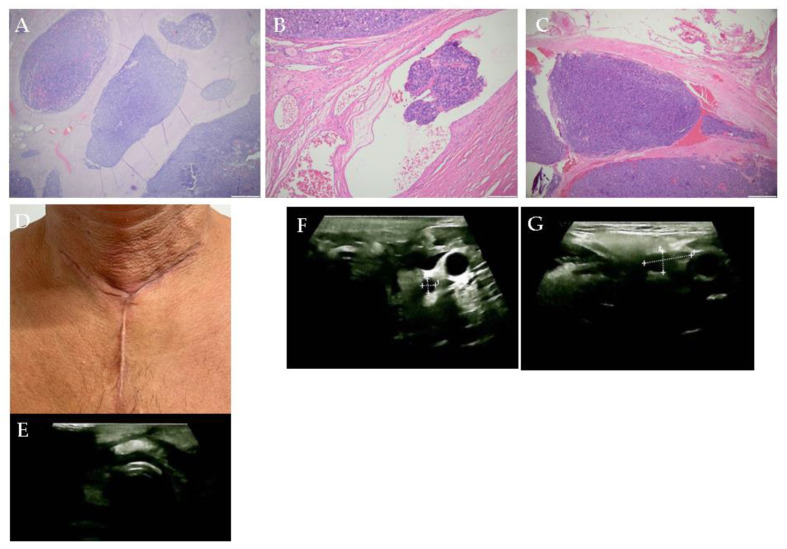
The pathological report confirmed poorly differentiated multifocal thyroid carcinoma (with an insular, solid, and trabecular pattern), which was developed against a background of papillary carcinoma, presenting isolated psammoma bodies, dystrophic calcifications, surrounding fibro-adipose and striated muscular tissue infiltration (pT3b), frequent intravascular tumoral emboli in the lymphatic vessels (L1) and venular vessels (V2), unapparent peri-neural invasion (Pn0), and surgical resection margins with carcinomatous tumor infiltration. (R1)—pT3b pN0 pM1; L1; V2; Pn0; R1; and stage IVB thyroid carcinoma. (Histological analysis: (**A**). Hematoxylin—eosin staining; magnification 2×. (**B**). Vascular invasion, magnification 10×. (**C**). Venular invasion with 4× magnification). Immunohistochemistry showed positive thyroglobulin in the tumor cells, negative calcitonin, chromogranin A, and Ki67 of 20%. Tissue samples were fixed in formalin and included in paraffin for the sequencing of exon 15 of the *BRAF* gene [black PREP FFPE DNA Kit (Analytik Jena)]. The positive *BRAF V600E* (NM_004333.6(BRAF): c.17997 > A (p. Val600Glu) variant was confirmed in the tumor tissue. The patient developed post-operative hypoparathyroidism-related hypocalcemia (Table S1), requiring oral and intravenous calcium supplementation in addition to vitamin ((**D**). Post-cervicotomy, early image of the scar). Following the surgery, an anterior neck ultrasound showed small bilateral hypoechoic remnants and intense edema. [Anterior neck ultrasound: (**E**). Pre-tracheal highly inhomogeneous area suggestive of edema (transverse plane). (**F**). Adenopathy of 0.47 by 0.5 cm in the lower part of the left latero-cervical area (transverse plane). (**G**). Left submandibular adenopathy of 1.7 by 0.73 cm (transverse plane)].

**Figure 5:**
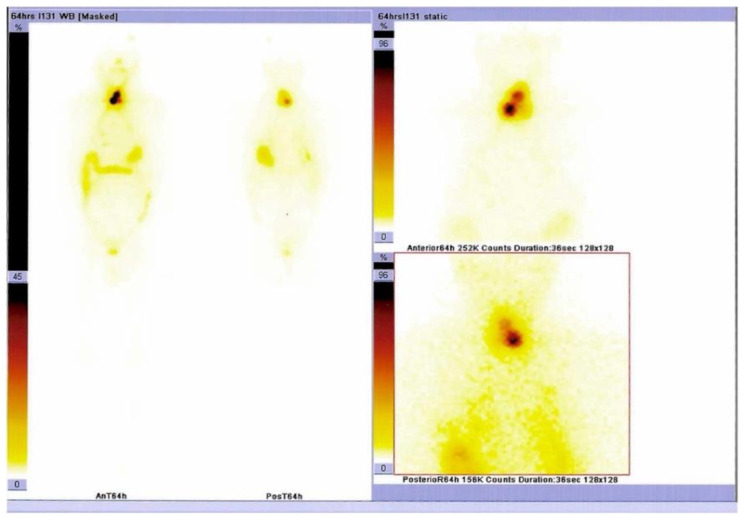
Serum thyroglobulin levels were above the maximum detectable value (>550 ng/mL). (Table S1). The patient was referred for radioiodine therapy and received 200 mCi for 6 weeks post-operatively (without levothyroxine replacement). Whole-body scintigraphy was performed at the moment before radioiodine was administered and showed increased radiotracer uptake at the thyroid remnants and pre-tracheal levels.

**Figure 6:**
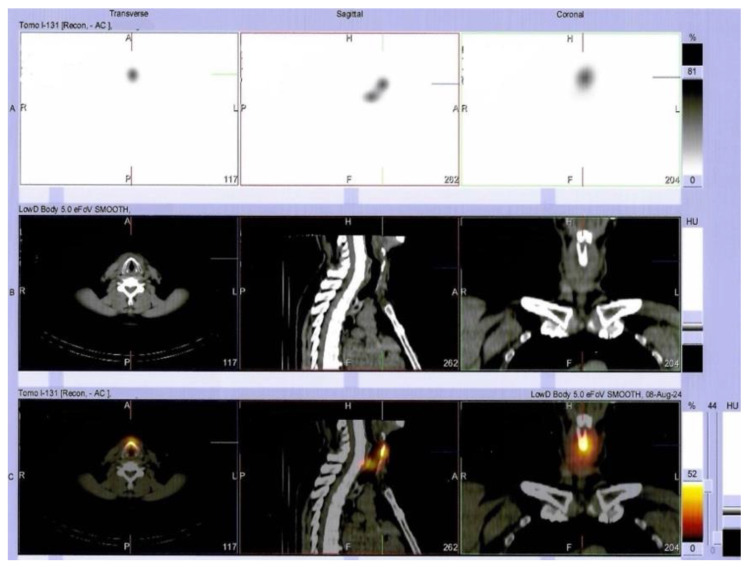
Additionally, single-photon emission-computed tomography combined with CT (SPECT/CT) was performed (immediately after radioiodine administration) and confirmed areas of intense uptake at the level of both the thyroid lobes and pre-tracheal, corresponding to iodine fixating thyroid remnants that during scintigraphy (upper row (**A**): SPECT image; middle row (**B**): CT image, lower row (**C**): SPECT/CT fusion image).

**Figure 7:**
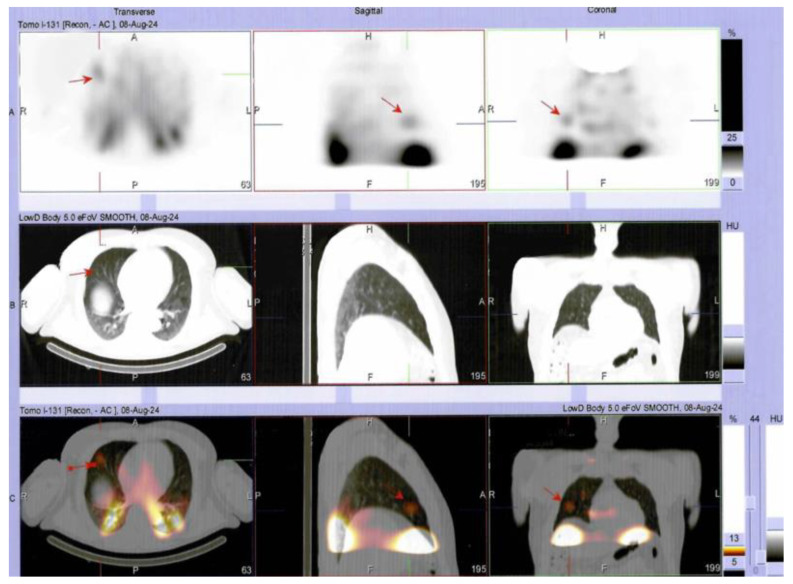
Additionally, SPECT/CT findings revealed areas with moderate uptake in the right pulmonary parenchyma, suggesting lung metastases (upper row (**A**): SPECT image; middle row (**B**): CT image, lower row (**C**): SPECT/CT fusion image).

**Figure 8:**
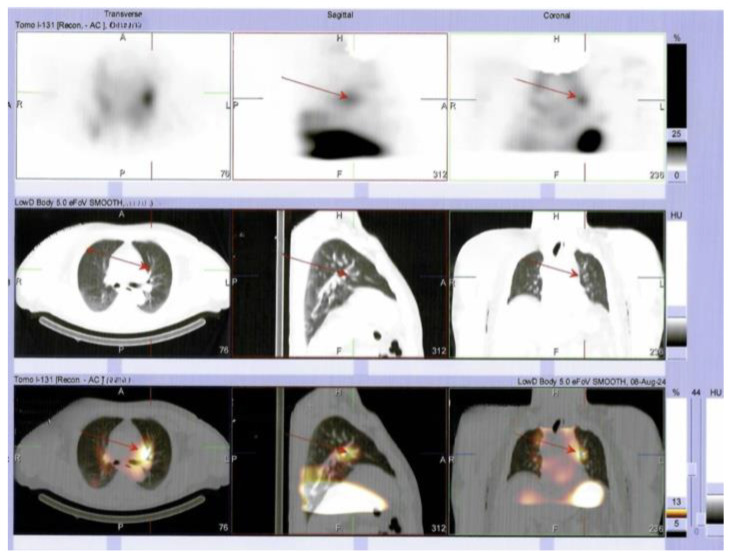
SPECT/CT also showed areas with moderate uptake in the left pulmonary parenchyma, suggesting lung metastases. Furthermore, the patient continued TSH suppressive therapy with daily levothyroxine. A serial thyroglobulin check-up is mandatory in order to decide on additional radioiodine therapy. In the case of radioiodine resistance, the initiation of tyrosine kinase inhibitors (TKIs) is taken into consideration. Poorly differentiated carcinoma belongs to a less common category of aggressive thyroid carcinomas (in addition to differentiated high-grade and anaplastic types). This group affects < 10% of all thyroid malignancies as opposed to most cases of follicular cell-derived cancers, which are papillary and follicular. This histological type is less understood, and the true incidence rate greatly varies (from 1 to 2% to 10% for all thyroid cancers). One of the causes of this is a large spectrum of histological patterns that often lead to misdiagnosis (e.g., insular, trabecular, and solid patterns, as found in this case; generally, oncocytic types are associated with worse prognosis), as well as a complex landscape of molecular characterization. This topic is cutting-edge research at the moment, as part of a multimodal strategy of management, since TKI, checkpoint inhibitors, and anti-BRAF medication represent novel therapeutic strategies [1,2,3]. As seen here, local (capsular, lymphatic, vascular) and regional invasion are reported in 50–85% of these cases; 21% of the subjects present distant metastases at first presentation. The rate of lymph node metastases varies between 12% and 73%. Male sex, advanced age, lack of tumor encapsulation, vascular invasion, and extra-thyroid spreading were found to be poor prognostic factors. The overall survival rate was 55.8 months; 5-year survival ranged from 65.71% to 73.8%, indicating a more severe behavior compared to differentiated high-grade carcinoma, and less severe behavior than anaplastic cancer. A tumor size >4 cm is considered a predictor of radioiodine refractoriness [1,4,5,6,7]. Most common genetic/molecular anomalies involve *TERT*, *RAS*, and *BRAF,* but rarely *TP53*, *PTEN,* and *PIK3CA*. Among aggressive thyroid cancers, anaplastic types have the highest correlation with mutational burden, but poorly differentiated types have *RAS*- and *TERT*-driven malignancies in almost half of cases, while other mutations are reported (each) in 5% to 15% of patients, depending on the study. The *BRAF V600E* variant excludes RAS variants but might co-exist with *TERT*. *RAS* and *TERT* are the most frequent gene anomalies associated with a poor response to radioiodine therapy, while combinations (*BRAF* + *TERT* or *TERT* + *PIK3CA*) have the worst outcomes [1,8,9,10]. Moreover, the data regarding retrosternal presentation of the goiter underlying this histological type are scarce, and it is still an open discussion as to whether this anatomic interplay might contribute to a more severe prognosis. Generally, a substernal/retrosternal goiter, representing gland enlargement below the thoracic inlet (>50% of the thyroid mass), is rarely found nowadays. Controversies concerning the terminology of the cervico-mediastinal goiters and the retrosternal extension of an orthotopic thyroid mass are still present. The distinction between a retrosternal goiter and an ectopic mediastinal thyroid is determined by the presence of connective tissue with the eutopic gland and the composition of blood supply, which is independent from the vessels of the eutopic gland in the mediastinal (ectopic) presentation [11,12,13,14,15]. In this study’s case, one-third of the tumors developed substernal and associated vessels originating from the cervical region. The “Y” cervicotomy allowed adequate access and is part of a local protocol in the high-volume Thoracic Surgery Department [11], while the surgical procedure/technique depends on the surgeon’s experience. The neck “Y” incision was performed by adding an extra 5 cm downward (over the manubrium) to the vertical incision compared to the classical Kocher incision. This combined incision has a vertical component that enables extended access to the upper thoracic inlet without sectioning the sternum. Furthermore, it provides access for emergency sternotomy, if necessary, and it allows the surgeon to follow the trajectory of the recurrent laryngeal nerve upwards from the superior mediastinum towards the neck, ensuring the most effective surgical approach. Of note, intraoperative local invasion of the left strap muscles had no CT scan correspondence. In some instances, a retrosternal goiter will require a sternotomy, particularly when the mediastinal vascular bundle is present or there is an invasion of the surrounding structures. In most cases, an enlarged mediastinal component descending below the aortic arch indicates a sternotomy. Notably, it is not always possible to see the vascular connections of the thyroid and the mediastinal vessels in a CT scan. In such cases, the decision for sternotomy is taken intraoperatively. If these vessels are present, a massive mediastinal hemorrhage might occur during the delivery of the mediastinal component, which is a blind maneuver. This is due to tearing of the mediastinal thyroid bundle. Generally, the transthoracic approach is associated with a 30% higher rate of morbidity than found after trans-cervical incision. Alternatively, a thoracoscopic-assisted trans-cervical approach, if feasible, might reduce the rate of post-surgery complications [12,13,14,15,16,17,18,19,20]. In our case study, the patient underwent a fast and complete recovery due to the surgeon’s optimal access to the superior thoracic outlet provided by the “Y” cervicotomy. There was no post-operative pain, with a good esthetic result and rapid hospital discharge. Radical excision of a poorly differentiated carcinoma (if feasible) has been found by some authors to be a good prognostic factor, and it represents a mandatory first step in management. On the contrary, incomplete resection is associated with a poor outcome [1]. Interestingly, despite tracheal deviation, the patient did not present any suggestive compressive neck signs or symptoms, except for cervical goiter palpation, which developed as a rapidly growing painless mass over a few months. Clinical exams can identify valuable clues for prompt intervention and access to thyroidectomy. Multilayered management in this distinct instance also includes additional diagnosis tools such as SPECT/CT, which confirmed the lung metastases bilaterally. To conclude, as clinical care points, awareness remains the key factor in the case of a poorly differentiated carcinoma presenting as a rapidly growing goiter with substernal extension in an apparently healthy adult. The surgical approach, while varying based on a surgeon’s skills, represents the mandatory step needed to ensure a better prognosis. In addition to a meticulous histological characterization, genetic/molecular features provide valuable information regarding the outcome and will further help the decision to use new anti-cancer drugs if tumor response upon radioiodine therapy is no longer achieved. Such a development is expected in this stage in addition to a *BRAF*-positive configuration.

## Data Availability

All the data are contained in this article.

## Supplementary Materials

The following supporting information can be downloaded at: https://www.mdpi.com/article/10.3390/diagnostics15162049/s1. Table S1. Endocrine evaluation (thyroid and parathyroid-related panel) showing normal thyroid function with mild vitamin D deficiency at baseline (pre-operatory); hypocalcemia due to hypoparathyroidism one week after surgery; as well as an extremely elevated blood thyroglobulin as post-surgery tumor marker of thyroid cancer).

## Abbreviations

The following abbreviations are used in this manuscript:

CT	computed tomography
FT4	free levothyroxine
PTH	parathyroid hormone
P1NP	procollagen type 1 N-terminal propetide
TSH	Thyroid-stimulating hormone
TgAb	anti-thyroglobulin antibodies
TPOAbs	anti-thyroperoxidase antibodies
TRAb	TSH-receptor antibodies
T4	thyroxine
SPECT/CT	single-photon emission computed tomography combined with CT
