# Supplementary material for: Multilayered Insights into Poorly Differentiated, BRAFV600E-Positive, Thyroid Carcinoma in a Rapidly Developing Goiter with Retrosternal Extension: From En “Y” Cervicotomy to SPECT/CT-Positive Lung Metastases
Source: Diagnostics (Basel). 2025 Aug 15;15(16):2049. doi: 10.3390/diagnostics15162049 (PMC12385848; doi:10.3390/diagnostics15162049)
Supplement: Supplementary file 1 [file diagnostics-15-02049-s001.zip › diagnostics-3808183-supplementary.pdf]

Table S1. Endocrine evaluation (thyroid and parathyroid-related panel) showing normal thyroid function with mild vitamin D deficiency at baseline (pre-operatory); hypocalcemia due to hypoparathyroidism one week after surgery; as well as an extremely elevated blood thyroglobulin as post-surgery tumor marker of thyroid cancer)

Variable	On admission	One week after thyroidectomy	One month after radioiodine therapy	Normal range
		Thyroid panel		
TSH (μIU/mL)	0.45	0.4	15.16*	0.35-4.94
FT4 (pmol/L)	11.88	19	13.79*	9-19
TPOAb (IU/mL)	1.24	1.23	1.40	0-5.61
TgAb (IU/mL)	62.99	20.16	18.87	0-115
TRAb (IU/L)	1.34	NA	1.09	0-1.75
Thyroglobulin (ng/mL)	NA	**>550**	**1487**	3.5-77
Calcitonin (pg/mL)	4.61	NA	<1	1-11.8
		Mineral metabolism and bone turnover markers		
Total serum calcium (mg/dL)	9.7	**6.7**	8.6**	8.4-10.2
Ionized calcium (mg/dL)	3.8	2.98	3.87**	3.8-4.8
Serum phosphorus (mg/dL)	3.08	5.57	3.1	2.5-4.5
25-hydroxyvitamin D (ng/mL)	17	22	34**	20-100
PTH (pg/mL)	65	**7.6**	33.89	15-65
Osteocalcin (ng/mL)	17.11	19.8	12.01	14-46
Alkaline phosphatase (IU/L)	67.6	73	57.5	35-129
P1NP (ng/mL)	31.41	49.85	16.99	20.25-76.31
Cross-Laps (ng/mL)	0.229	0.22	0.10	0.104-0.504

*under 100 μg of levothyroxine daily.

**under calcium 600 mg, cholecalciferol 2400 IU and alfacalcidol 1μg per day.

Abbreviations: FT4 = free levothyroxine; PTH = parathyroid hormone; P1NP = procollagen type 1 N-terminal propetide; TSH = thyroid stimulating hormone; TPOAb = anti-thyroperoxidase antibodies; TgAb = anti-thyroglobulin antibodies; TRAb = TSH-receptor antibodies (of note, P1NP, osteocalcin and alkaline phosphatase represent bone formation markers, and Cross-Laps represents bone resorption marker).
